# Individualized Transcriptional Resolution of Complicated Malaria in a Colombian Study

**DOI:** 10.3390/jpm8030029

**Published:** 2018-09-14

**Authors:** Mónica L. Rojas-Peña, Meixue Duan, Dalia Arafat, Lina Rengifo, Socrates Herrera, Myriam Arévalo-Herrera, Greg Gibson

**Affiliations:** 1 Center for Integrative Genomics, Georgia Institute of Technology, Atlanta, GA 30332, USA; 2 CAUCASECO, Cali, Colombia

**Keywords:** transcriptomics, RNAseq, gene expression, longitudinal profiling, *Plasmodium*

## Abstract

To evaluate whether recovery from complicated malaria follows a common trajectory in terms of immunological mechanism or, rather, is highly individualized for each patient, we performed longitudinal gene expression profiling of whole blood. RNA sequencing (RNAseq) was performed on blood samples obtained from eight patients on four consecutive days between hospital admission and discharge. Six patients were infected with *Plasmodium falciparum*, and two with *Plasmodium vivax;* one patient was a pregnant woman infected with *P. falciparum*, who was hospitalized for several weeks. The characterization of blood transcript modules (BTM) and blood informative transcripts (BIT) revealed that patients’ responses showed little commonality, being dominated by the balance of gene activity relating to lymphocyte function, inflammation, and interferon responses specific to each patient. Only weak correlations with specific complicated malaria symptoms such as jaundice, thrombocytopenia, or anemia were observed. The differential expression of individual genes, including transcripts derived from the human leukocyte antigen (HLA) complex, generally reflected differences in the underlying immune processes. Although the results of this pilot study do not point to any single process that might provide a target for complicated malaria treatment or prevention or personalized medical strategies, larger patient series and more extensive blood sampling may allow the classification of patients according to their type of response in order to develop novel therapeutic approaches.

## 1. Introduction

Malaria is the most prevalent parasitic disease worldwide with over 200 million clinical cases and about 445,000 deaths reported globally every year [1]. While most clinical cases are characterized by classical signs and symptoms such as fever, chills, headache, and malaise that rapidly disappear upon early diagnosis and prompt treatment, failure to recover quickly can lead to a more severe and complicated disease that usually requires hospitalized management [2,3,4,5]. In these cases, multiple organs can suffer irreversible damage, leading to multi-systemic failure and eventually to death [6]. In Colombia, a low-malaria-transmission country of Latin America, the most common complications include vascular obstruction, inflammatory processes, and damage to as well as cell death in various organs. Symptoms include jaundice, severe thrombocytopenia, liver and kidney injury, and acute anemia, with cerebral infection affecting a minority of patients [7,8,9,10]. It is not precisely known what mechanisms are involved in the progression to severe disease or recovery from infection, although it has been shown that multiple arms of the immune system are engaged [11,12,13]. In *Plasmodium vivax*, a significant modification of peripheral blood gene expression is first observed as parasites begin to appear in the blood stream 12 to 14 days after initial infection [14]. It involves both myeloid and lymphoid cell types, as well as inflammatory and interferon responses. No clear pattern of Th1, Th2, or Th17 engagement has emerged, and, although high parasitemia is the major factor influencing the extent of transcriptional divergence [15], these results have not yet produced a clear picture of the chain of immune responses that control the infection. Complement activation is critical for the removal of *Plasmodium*-infected red blood cells [16,17], but very little is known about how variation among individuals, and indeed within individuals across infections, results in responses that range from asymptomatic infection to severe and complicated disease leading to mortality.

Recently, RNA sequencing (RNAseq) has emerged as the preferred tool for gene expression profiling, since it provides an unbiased view of the abundance of both genes and exons [18,19]. Compared with microarray analysis, technical artefacts seem to be less prevalent, but its application to complex tissues still suffers from the problem that cell type-specific differential gene expression can be imputed only indirectly. Studies of semi-purified immune cell subtypes certainly increase resolution but are more expensive, necessarily biased towards cells of a priori interest, and there is always the concern that purification modifies gene expression. Consequently, bioinformatics approaches are evolving that allow the deconvolution of the abundance of cell types as well as the inference of the gene activity pathways that are most strongly affected in a disease or treatment [20]. In the near future, single-cell RNAseq [21] will allow the comprehensive comparison of gene expression variation among all cell types of a mixture, potentially obviating the need for bioinformatic deconvolution.

By applying a machine learning approach to over 30,000 peripheral blood profiles, Li et al. [22] identified about 300 “blood transcript modules” (BTM) enriched for gene activity, known targets of key regulatory factors, or specific biochemical pathways in dozens of immune cell types. The derivation of the first principal component (PC1) of each BTM provides a summary of the major gene ontologies that characterize individual samples. Similarly, Chaussabel and colleagues [23] found 28 modules that are conserved across datasets but characteristic of 11 different immune diseases. We reduced these to nine super-modules, or “blood informative transcript” (BIT) axes [24], after recognizing that there is a very high correlation between the PC1 scores in multiple cohorts of healthy people. Subsequent analysis of dozens of datasets, including the results presented below, confirmed that BTM cluster into no more than a dozen groups, each of which covaries with one of the BITs. Furthermore, we show here that, by utilizing single-cell RNAseq profiles, we can now also characterize T-lymphocyte activity as helper, cytotoxic, or natural killer (NK) cell-related, from whole RNA profiles.

The objective of this study was to pilot the use of longitudinal RNAseq profiling during treatment and recovery from complicated malaria to characterize the immunological pathways engaged in the resolution of the infection. Whole blood samples were obtained upon hospitalization and three more times over the next four days. Most patients were discharged on days 4 or 5, but one pregnant woman (CM02) remained sick and hospitalized for 49 days; she was followed for almost a month and suffered a malaria relapse 15 days after first hospital discharge. Specifically, in this study, we examined: (i) whether there is a distinct signature of complicated malaria upon hospitalization of a patient, (ii) whether there is a common pattern of recovery characterized by the engagement of particular BTM or BIT, and (iii) whether individualized profiles associate with particular symptoms of complicated malaria.

## 2. Materials and Methods

### 2.1. Study Design and Subject Recruitment

Regardless of age, sex, or ethnic group, eight malaria patients admitted to a third-level hospital with confirmed *Plasmodium falciparum* (*n* = 6) or *P. vivax* (*n* = 2) were enrolled. The three study recruitment sites are located on the Pacific coast region of Colombia (Choco, Valle del Cauca, and Nariño Departments) and were sampled over the course of complicated malaria episodes in 2015. Clinical complications attributable to malaria infection were recorded, and complicated malaria was defined on the basis of existing guidelines of the World Health Organization (WHO) and the Colombian Ministry of Health [25,26], requiring clinical severity and one or more clinical conditions such as anaemia (Hb < 7 g/dL in adults and children), renal dysfunction (serum creatinine > 1.5 mg/dL), severe thrombocytopenia (≤20,000 platelets/μL), and hyperparasitaemia (>50,000 parasites/μL). The patients were medically monitored daily until discharge upon recovery from their clinical complications. All patients provided written informed consent for participation before enrolment, and the study protocol was approved by the Institutional Review Board (IRB) for the Malaria Vaccine and Drug Development Center (CECIV Approval letter 1.0) with DMID protocol 13-0056 on 14 February 2014, also amended on 1 October 2014 (Version 2.0) and 27 January 2016 (Version 3.0). In addition, the Georgia Institute of Technology IRB provided subsequent approval for genomic analyses on 4 April 2014, Amendment #1 to protocol H13495.

### 2.2. Blood Sample Collection

All study participants were bled by arm venipuncture at four time points during hospitalization, with the exception of the pregnant woman (CM02), who had two additional bleedings during a second hospitalization, because of a parasite relapse a few weeks after her first hospital discharge. Blood (~1 mL) was collected into Tempus tubes (Thermo-Fisher Scientific, Waltham, MA, USA), which preserve whole blood RNA indefinitely at 4 °C [27]. mRNA was extracted using Tempus Blood RNA tube isolation kits, following the protocol provided by the manufacturer, and the RNA quality was determined using the Agilent Bioanalyzer RNA Integrity score (RIN) (Agilent, Santa Clara, CA, USA) [28].

### 2.3. Whole Transcriptome Sequencing (RNAseq) and Preprocessing of the Data

Library preparation for RNAseq was performed using the Illumina TruSeq Stranded mRNA Sample Low-Throughput (LT) preparation protocol [29]. Short read sequencing was performed in rapid run mode with 10 samples per lane on an Illumina HiSeq 2100 (Illumina, San Diego, CA, USA) at Georgia Tech, generating 100 bp single-end libraries with an average of ~30 million single-end reads per sample.

The Tophat2 package [30] was used to align the reads to the human genome hg19/GRCh37, followed by UCSC reference annotation and HTSeq [31] to estimate the transcript abundance. The expression of known transcripts was calculated as counts per million (cpm) and filtered by cpm equal or greater than 1 in at least three of the samples; a total of 13,958 genes passed this criterion and were scaled on the log 2 scale by trimmed mean of the m-values (TMM) normalization in edgeR [32].

### 2.4. Analysis of RNAseq Data

#### 2.4.1. Principal Variance Component Analysis

We performed principal variance component analysis (PVCA) to assess the overall contributions of patient, time-point, gender, and *Plasmodium* species to the transcriptional variance using the PVCA package in R v3.3.1. The percentile value for the minimum amount of the variance explained by the PC was set to 75%, and the average contributions of each covariate were computed as the average contribution to each PC weighted by the variance explained by the PC. PC1 mainly captures inter-individual variation, whereas PC2 and PC3 derive from a mixture of sources, with a strong influence of the parasite species.

#### 2.4.2. Modular Analysis

Blood transcript modules [22] and BIT [24] analyses were performed by computing the PC1 of all the genes in each module or the top genes most closely correlated with each BIT axis. Standard analysis of variance (ANOVA) was then used to compare these scores among treatment classes, and two-way hierarchical clustering was computed using Ward’s method in JMP Genomics version 8 (SAS Institute, Cary, NC, USA). The Module and Axis scores for each individual at each time point are listed in Supplementary Table S1.

In order to refine the contributions of NK cells, T-helper cells, and T-cytotoxic cells, we identified the top 10 genes differentially expressed between these three cell types in the single-cell RNAseq dataset reported in reference [21]. The first principal component for each of these three sets is, as expected, highly correlated with Axis T, but the residuals of the regression of each PC1 on Axis T provide a measure of inflation or repression of the gene activity in the three cell types.

#### 2.4.3. Differentially Expressed Gene Calling and Gene Ontology Analysis

One-way ANOVA analysis between each patient and the remaining patients was conducted to detect patient-specific differentially expressed genes, using the following arbitrary *p*-value thresholds to identify approximately 50 to 150 genes per individual: *p* < 10^−4^: CM09, CM14, CM44, and CM52, detecting 35, 48, 101, and 53 genes, respectively; *p* < 10^−5^: CM41 detecting 55 genes; *p* < 10^−6^: CM04, CM05, detecting 163 and 86 genes, respectively; *p* < 10^−8^: CM02, detecting 242 genes (the larger sample size for this individual increased the statistical power, resulting in more detections).

After removing duplicated differentially expressed genes (DEGs), the TMM expression levels of the 781 genes listed in Supplementary Table S2 were subjected to two-way hierarchical clustering using Ward’s method, and eight differentially expressed gene modules were observed. Functional annotation of these modules by gene ontology and other categories was assessed using the ToppFun tool of the ToppGene suite online [33]. A full list of results is provided in Supplementary Table S3.

## 3. Results

### 3.1. The Individual Is the Major Source of Variation as Patients Recover from Infection

The characteristics of the study participants are shown in Table 1. The age range was 3 to 69 years (mean 34 years), with four individuals of each sex. Five patients were from the mid-Pacific lowland region of Chocó (CH), one from the Valle del Cauca (VC) region, and two from the southwest Pacific coast region of Nariño. Clinical presentations included respiratory distress, jaundice, and oral intolerance to drug administration, while blood laboratory tests revealed severe thrombocytopenia and/or anemia, as well as renal and hepatic dysfunction, in several instances; as expected, parasitemia was lower in the two *P. vivax* malaria cases than in four of the six *P. falciparum* cases [10].

The PVCA of the full dataset consisting of 40 RNAseq samples from the eight complicated malaria cases revealed that 54% of the variation was recorded among individuals, 12% between the two *Plasmodium* species, 10% between males and females, and <1% between days. The contributions of each source of variation to individual PC are listed in Supplementary Table S4. The first principal component mainly captures inter-individual variation, whereas PC2 and PC3 derive from a mixture of sources and include strong influences of the parasite species. Although there was a considerable variation across days within individuals, this analysis clearly showed that overall there was not a common profile of resolution whereby patients moved from an acute profile at hospitalization to a healthier profile at discharge. It is unclear whether the parasite and sex components were significant, given that just two patients were infected with *P. vivax*, and that, in large samples of healthy individuals, sex accounts for only a few percent of blood gene expression variation (e.g., [24,34]). In order to contrast complicated with uncomplicated malaria, we added another 24 samples to the dataset, consisting of 12 Colombian individuals previously subjected to experimental malaria infection, who were sampled before and after infection [14]. All but one of the complicated malaria samples clustered with these experimentally infected samples (data not shown), all of which were immediately resolved following standard malaria treatment, and none were associated with severe symptoms. Consequently, there is no evidence that the complicated cases are characterized by a distinct profile of infection that is qualitatively different from uncomplicated malaria.

### 3.2. Modular Analysis Resolves the Major Immune Activities Engaged in Each Individual

To gain insight into the immunological pathways that vary within and among individuals, we next performed BTM and BIT analysis by generating the PC1 for each set of BTM module genes or the 10 characteristic BIT axes (see Materials and Methods for details). An initial nomenclature considered Axis 1 through Axis 9 [24]; however, for a better interpretation of results, we recently [35] renamed the Axes T, B, N, R, I, G, and C for T cell, B cell, Neutrophil, Reticulocyte, Interferon response, General, and Cell cycle, corresponding to Axes 1, 3, 5, 2, 7, 4, and a newly discovered Axis 10, respectively. The former Axes 6, 8, and 9 were excluded from the analysis because we do not know their biological function. Axis C is enriched for genes involved in mitosis and cell cycle regulation, and the PC1 score for this Axis is very similar to that of multiple BTM annotated to cell cycle functions.

**Figure 1:**
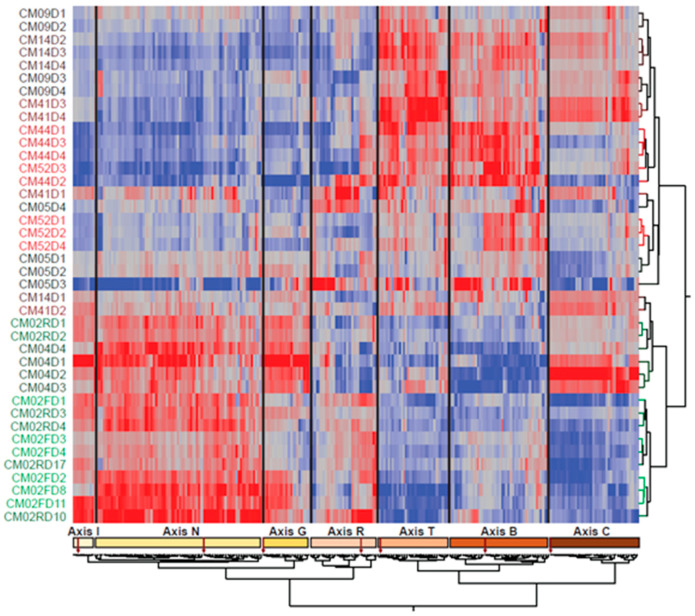
Two-way hierarchical clustering of blood transcript modules (BTM) and blood informative transcripts (BIT) by individual and time. Each row represents one patient on the day indicated by the last number of the label, and each column is the principal component (PC1) score for a BTM, with co-clustered BIT PC1 highlighted. The red-to-blue scale indicates positive to negative scores. The figure highlights the tendency of the samples to cluster by individual and provides an overview of the axes of variation shared by particular patients.

Two-way hierarchical clustering of the 253 BTM and 7 BIT scores in Figure 1 with individual samples as rows revealed a very clear structure, whereby the individual samples all tended to cluster with one another, and the BTM fell into six clusters indicated along the bottom, each of which included one BIT axis as indicated. The leftmost cluster actually included both Axis I and Axis N (though they are in distinct sub-clusters, as shown by the lower dendrogram), indicating that inflammation and interferon response were tightly coordinated in this dataset. The Reticulocyte Axis is right next to BTM173 (erythrocyte differentiation) and BTM171 (heme biosynthesis) and immediately adjacent to the two platelet activation modules (BTM32_0 and BTM32_1). All five patients who reported severe thrombocytopenia had at least two time points with a standard deviation more than one unit below the mean for these two modules (although CM52 had the two lowest scores). Intriguingly, on the right of the figure, all of the cell cycle modules and Axis C quite cleanly distinguished four patients with consistently low mitotic activity (CM02, CM05, CM44, and CM52) from the remainder. Three of these patients experienced respiratory distress, as did CM09, whose cell cycle expression level was intermediate. See Supplementary Table S5 for the identities of the BTM in each cluster.

**Figure 2:**
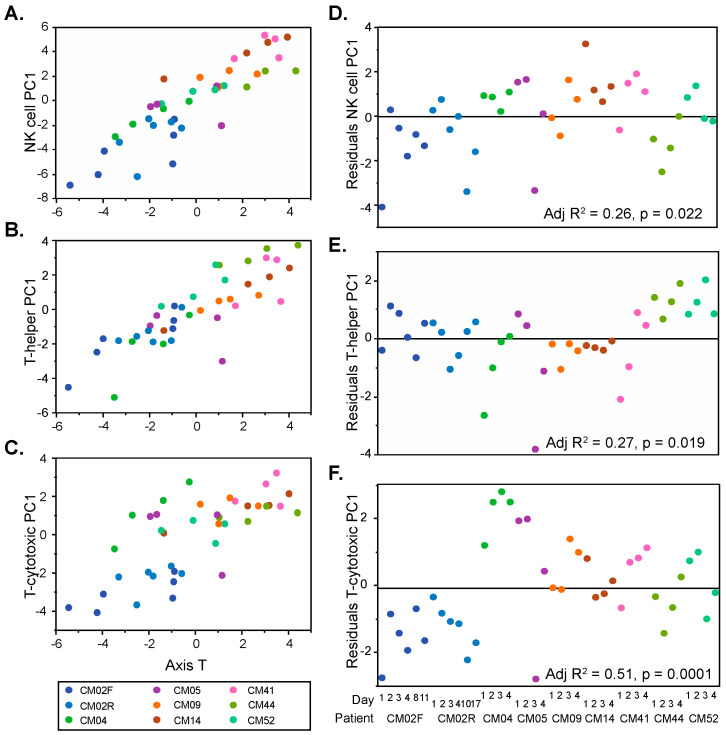
Lymphocyte activity inference in each individual. Each plot (**A**–**C**) shows how a PC1 based on natural killer (NK) cell, T-helper cell, or T-cytotoxic cell specific transcripts correlates with Axis T and varies by individual, indicated by different colors. Graphs (**D**–**F**) plot the residuals of the linear regressions by individual, highlighting patient-specific lymphocyte activity. *p*-values and adjusted R^2^ estimates were calculated through an analysis of variance (ANOVA).

The B and T cell activity Axes clustered with 78 BTM annotated to various aspects of lymphoid signaling and activity. We attempted to further resolve the immune cell contributions by taking advantage of recently reported single-cell RNAseq results from whole blood [21], which document dozens of genes significantly elevated in NK cells, cytotoxic T cells, or helper T cells (see Materials and Methods). Figure 2A–C show that PC1 for each of these gene sets is highly correlated with Axis T, explaining the lack of resolution of the sub-types in Figure 1. However, the residuals of the regressions significantly differed among patients, as shown in Figure 2D–F. Our interpretation is that the individuals with positive residuals had a higher abundance of the genes representative of that cell type, likely implying the elevated abundance of the cell type itself. By this reasoning, CM04, CM14, and CM41 had consistently elevated NK cell activity, CM44 and CM52 had elevated helper T cell activity, and CM04 had very high cytotoxic T cell activity, whereas CM02 showed a remarkably low activity of that component.

**Figure 3:**
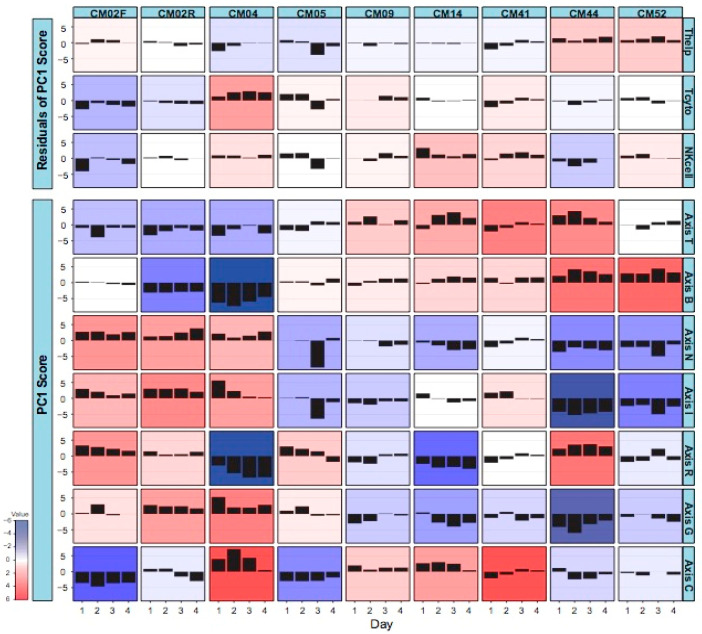
Standardized change in BIT by individual and time. Each box shows the difference between the sample PC1 score and the sample mean for the indicated individual from Day 1 to Day 4 of sampling. Red implies strong elevation, and blue strong reduction in the expression of genes in the Axis.

The time course of BIT Axis scores for each patient is summarized in the matrix of histograms presented in Figure 3. The results strongly imply that each patient confronted the malaria infection with a quite different immune response. In short, the following patterns emerged. Table 1 lists the clinical presentation features for each patient, but there was no clear relationship between the clinical data and the gene expression data, except for a few cases mentioned below.

- CM02, who presented with severe anemia clinical manifestations and relatively low parasitemia, had generally low cytotoxic T-lymphocyte and natural killer cell activity, which may explain her failure to resolve the infection. She also had particularly low mitotic activity during the initial infection and low B cell function during the relapse. All this was compensated by elevated inflammatory and interferon responses.
- CM04, who experienced renal and liver failure, also had a notable inflammatory response and low B cell activity, but this was offset by high cytotoxic T cell and mitotic activity. His reticulocyte numbers were relatively low, consistent with the absence of anemia, and his interferon response settled down upon hospitalization.
- CM05, with severe thrombocytopenia, was notable for a dramatic drop in inflammation and interferon signaling specifically on day 3 and for a relatively high cytotoxic T cell function on days 1 and 2.
- CM09, with severe thrombocytopenia and high parasitemia, had a rather stable profile without any dominant characteristics, perhaps correlating with his less complicated disease or with his age (69), which suggests that he may have built up more tolerance to malaria than the younger patients.
- CM14, with hepatic failure and severe thrombocytopenia, was notable for very high NK cell signatures and relatively low reticulocytes.
- CM41, who experienced severe thrombocytopenia and jaundice, presumably due to liver dysfunction, had relatively high NK cell and cytotoxic T cell activity, as well as elevated cell cycle gene expression, similar to CM14.
- CM44 and CM52, both infected with *P. vivax*, had similar transcriptome profiles. CM52 was a 3-year-old boy at the time of this episode of malaria and was mainly hospitalized for respiratory distress. CM44 presented a more extreme condition (severe thrombocytopenia, jaundice, and renal injury) with respect to her combination of low inflammation (perhaps related to a relatively low parasitemia) and particularly high B cell and helper T cell activity.

### 3.3. Patient-Specific Gene Expression Reinforces the Conclusions of the Modular Analysis

**Figure 4:**
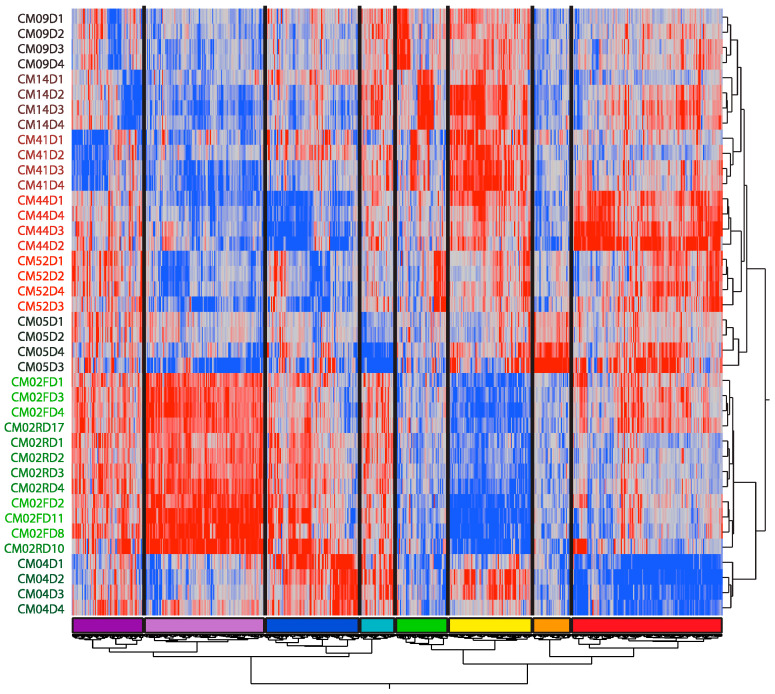
Two-way hierarchical clustering of differentially expressed gene (DEG) by individual and time. Each row represents one patient on the day indicated by the last number of the label, and each column is the standardized expression of a DEG in ANOVA contrasting each individual with the remaining sample. The figure highlights clusters of genes that tend to be patient-specific, but shows (like the BTM analysis) that these are to some extent shared by pairs of patients. The colored bar at base indicates clusters discussed in the text.

Given that each patient showed a relatively unique modular pattern during hospitalization, we next asked whether their gene expression patterns followed patient-specific trajectories. To do so, we identified patient-specific DEGs using one-way ANOVA (see Materials and Methods). After removing duplicated DEGs, we obtained 781 DEGs in total and performed two-way hierarchical clustering, observing eight DEG modules (Figure 4). Gene ontology functional enrichment analysis of the DEGs within each module revealed striking concordances with the modular analysis (Supplementary Table S3). It is also notable that with this mode of patient-specific gene expression analysis, the four samples for each patient always clustered together.

The expression of the red cluster genes was high in CM44 and CM02 during the first three days, and in CM05 on days 3 and 4, but low in CM04. Gene ontology analysis revealed enrichment for erythropoiesis and B cell differentiation markers, which was concordant with the modular analysis Axis R and Axis B variation patterns reported above. Similarly, gene expression in the yellow module was high in CM41, CM14, CM44, and CM09 but low in CM02, which, as gene ontology analysis suggested, may indicate gamma-delta T cell deficiency and mitochondrial dysfunction. Once again, this agreed with the Axis T of the modular analysis. Additionally, gene expression of the dark blue module, which is related to type I interferon signaling, was upregulated in CM02, CM04, and CM41 on days 1 and 2 but downregulated in CM44, matching the Axis I pattern of the modular analysis. Furthermore, gene expression of the light purple module was high in CM02, relatively high in CM04, but low in other patients. Gene ontology analysis implies that this module is related to neutrophils and inflammation, in line with the Axis N pattern of the modular analysis.

Even though the BIT and BTM patterns were validated by the modular analysis, a small number of more detailed patient-specific results were also revealed by the DEG analysis. For example, gene expression of the orange module was high in CM05 but low in other patients. Ten BTM related to myeloid dendritic cell activation were also upregulated in this patient.

### 3.4. Variable Expression of HLA Complex Genes among Patients

**Figure 5:**
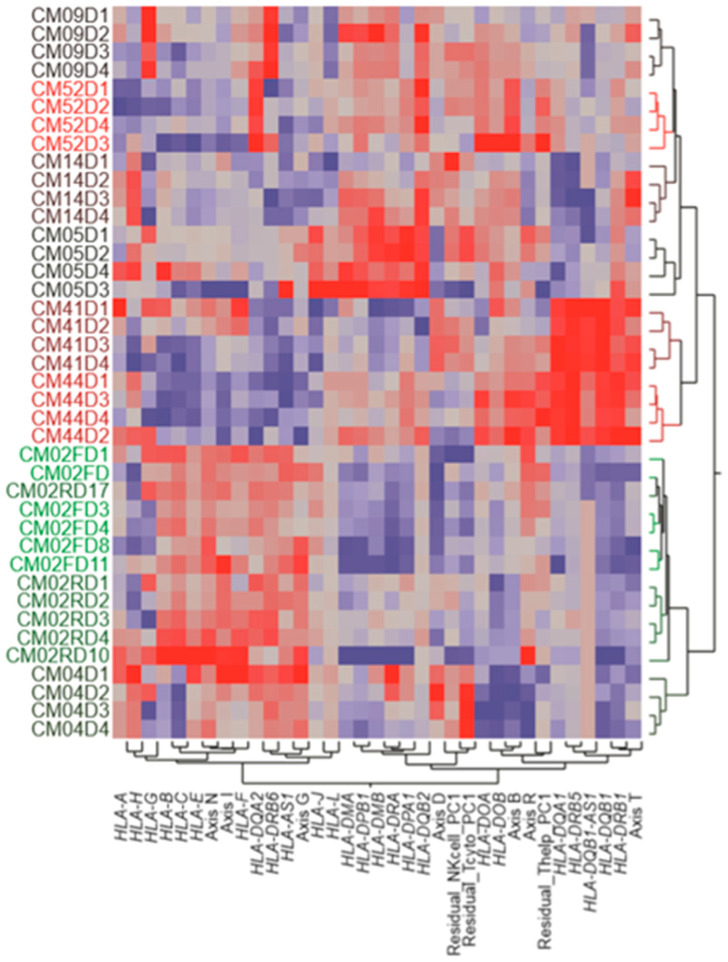
Two-way hierarchical clustering of HLA complex gene expression by individual and time. Each row represents one patient on the day indicated by the last number of the label, and each column is the standardized expression of the indicated HLA gene or lymphocyte activity estimate. The figure highlights three clusters of co-expression, namely, HLA class I to the left and two HLA class II clusters in the middle and right of the grid.

Since the human leukocyte antigen (HLA) complex is known to play an important role in the processes of protection against intracellular pathogens, including the immune response during malaria infection, we also examined the collective regulation of 25 HLA genes and their relationship to the BIT Axes (Figure 5). Overall, three clusters of co-regulated HLA expression were observed. The HLA class I genes, *HLA-A* to *HLA-H*, were all relatively highly expressed in CM02 and CM04, the two patients with the strongest inflammatory response. In addition, three individuals displayed an unusually high expression of single class I genes, namely, *HLA-G* in CM09, and *HLA-H* in CM14. Two class II genes were also notably uniquely elevated in these two individuals, i.e., *HLA-DRB6* and *HLA-DQA2*, respectively. It is not known whether these specific patterns may have influenced the complicated course of the disease.

Regarding the expression of HLA class II genes, these showed relatively low levels in the two patients with high neutrophil and interferon activity but separated into two sub-groups. One included *HLA-DMA*, *HLA-DMB*, *HLA-DPA1*, *HLA-DPB1*, *HLA-DRA*, and *HLA-DQB2*, the expression of which was particularly high in CM05, CM09, and CM52, all of whom were among the high inflammatory response individuals. This suggests a trade-off between inflammation and HLA-class I and class II gene expression. The second sub-group included *HLA-DQA1*, *HLA-DQB1*, *HLA-DRB1*, and *HLA-DRB5*, whose expression was notably elevated in CM41 and CM44, two individuals with relatively high T cell activity in general but otherwise contrasting profiles. Larger sample sizes will be required to ascertain whether and how HLA class II gene expression is associated with complicated malaria.

## 4. Discussion

Given that malaria is a blood stream infection, recovery from which requires elimination of parasite-infected red blood cells, our expectation was that longitudinal transcriptome profiling would reveal common regulatory signatures of engagement of one or more arms of the immune system. At least, elevated reticulocyte development and reduced engagement of innate and interferon-mediated responses, as patients move from acute clinical manifestations to healthy condition and release from hospital, would be expected upon recovery. However, while there were signs of such responses in a majority of the patients, the timing and strength of the signals revealed by Axes R, N, and I were notably variable among patients. No other strong signatures, for example of engagement of B cells, a subset of T cells, or perhaps a pathway such as antigen processing, were observed across the dataset. Rather, the overwhelming pattern was one of highly individualized profiles of immune-related recovery. The dominant variance component in the dataset was between-individual effects, and each person displayed a personal trajectory of recovery.

Such individualization is reminiscent of our findings in healthy individuals [36]. In that study, we found, by contrasting the RNAseq profiles of a dozen of individuals at three six-month intervals, that all subjects consistently clustered together, whether considering the major PC of the entire transcriptome or the Axis scores. This gave rise to the notion of an “omic personality”, a consistent profile of gene expression over time that reflects the baseline healthy status of an individual [36]. This baseline expression is quite likely to have a major influence on the recovery profiles.

Similarly, microarray-based profiling of lupus erythematosus patients [37] discovered at least eight dominant trajectories of gene expression over time. Clustering transcripts on the basis of their correlation with various clinical traits of relevance to pathology, including the systemic lupus erythematosus disease activity index (SLEDAI), showed that some patients had a predominantly B cell-related pattern, while, in others, reticulocytes, NK cells, or T cells seemed to produce the strongest correlations. Despite the well-documented interferon response seen in lupus, a plasmablast signature present in most patients, and neutrophilia associating with nephritis, it was the personalized nature of the immune gene expression monitoring that dominated the analysis.

These observations raise the question of whether baseline peripheral blood profiles might be predictive of the course of a disease or provide personalized information regarding the best course of the curative treatment. The lupus study was ambiguous on this point, and, despite trends such as association of low cell cycle activity with respiratory distress, our pilot study is too small to make any definitive statements. What is clear is that the different pairs of patients with high B cell (CM44 and CM52), NK cell (CM14 and CM41), or cytotoxic T cell (CM04 and CM05) activity did not share complicated malaria attributes to the exclusion of other patients, so a close matching of immune profile to clinical presentation is unlikely.

Another possible source of patient-specific clinical presentation is the aberrant expression of one or a handful of genes that lead to a complicated course rather than to a standard resolution. We noted several instances of divergent expression of *HLA* or other genes of interest, but samples from several thousand patients will be required to establish any statistical association with a phenotype. Most of the divergent expression was due to an individual having the highest or lowest values for the relevant axis of variation, but it is likely that mutations and segregating regulatory variants also contribute for specific genes. The individuals CM09, CM14, and C52, whose overall profiles had relatively normal axis scores, also had divergent *HLA* gene expression levels, and these genes may be involved in mediating aspects of the complicated course of disease.

It is not known what causes the strong patterns of covariance of gene expression. Some of it is due to the differential abundance of contributing lymphoid and myeloid cell types, but immune cell type abundance is known to have low heritability [38,39]. Aspects such as interferon response, cell cycle regulation, and general cellular metabolism are likely affected by multiple immune cell types, and the longitudinal consistency of the corresponding axis scores suggests a substantial trans-acting regulatory component. The interaction of the immune system with the parasite would significantly contribute at multiple stages of the infection cycle, from parasite entry and development in the liver cells to parasite multiplication during blood infection. *Plasmodium* antigens likely induce immune responses, which engage all of the major T cell subsets, possibly modulating the immune response, once the acute clinical phase of infection commences. Although, in malaria, the immune system is primed upon sporozoite injection by a mosquito under the patient’s skin, it is only during the parasite development in the blood stream that the clinical manifestations appear. Whether or not this knowledge can be used to understand and eventually manipulate the immune responses in a personalized manner is unclear but will likely require longitudinal studies involving thousands of patients across diverse infectious and complicated disease phenotype classes.

## Figures and Tables

**Table 1:** Patient demographic, clinical, and laboratory information.

Subject	Site	Sex	Parasite *Spp*/Counts	Clinical Criteria ^§^	Laboratory Criteria	Age	Hospital Days
CM09	Valle del Cauca	M	*P.f.*/25.1	OI, RD	ST	69	5
CM14	Chocó	M	*P.f.*/19.6	OI	Hep, ST, MH	37	5
CM41	Chocó	F	*P.f.*/142	OI, Jau	ST	49	4
CM44	Chocó	F	*P.v.*/5.7	OI, Jau	MH, RI, ST	25	5
CM52	Chocó	M	*P.v.*/5.7	RD	None	3	4
CM05	Chocó	F	*P.f.*/11.6	RD	ST	38	4
CM02 *	Chocó	F	*P.f.*/5.2, 4.3	RD	SA	28	11, 18
CM04	Nariño	M	*P.f.*/20.8	OI	RI, Hep	22	4

* 25-week pregnant, two visits. **Parasite *spp*:** *P.f*. = *Plasmodium falciparum*; *P.v.* = *Plasmodium vivax*. **Counts:** Parasites × 1000/μL. ^§^ Clinical criteria OI = oral intolerance, Jau = jaundice, RD = respiratory distress. Laboratory criteria ST = severe thrombocytopenia, SA = severe anemia, MH = macroscopic hemoglobinuria, RI = renal injury, Hep = hepatic failure.

## Supplementary Materials

The following are available online at https://www.mdpi.com/2075-4426/8/3/29/s1, Supplementary Table S1: BTM Module and BIT Axis scores. Supplementary Table S2: List of Differentially Expressed Genes. Supplementary Table S3: Gene Ontology Analysis. Supplementary Table S4: Principal Variance Components. Supplementary Table S5: BTM in each Cluster of DEG.
