# Correction: Rojas-Peña et al. Individualized Transcriptional Resolution of Complicated Malaria in a Colombian Study. *J. Pers. Med*. 2018, *8*, 29

**DOI:** 10.3390/jpm14101060

**Published:** 2024-10-14

**Authors:** Mónica L. Rojas-Peña, Meixue Duan, Dalia Arafat, Lina Rengifo, Socrates Herrera, Myriam Arévalo-Herrera, Greg Gibson

**Affiliations:** 1 Center for Integrative Genomics, Georgia Institute of Technology, Atlanta, GA 30332, USA; monicarojasp@gmail.com (M.L.R.-P.); mduan31@gatech.edu (M.D.); darafat.gt@gmail.com (D.A.); 2 CAUCASECO, Cali, Colombia; lrengifo@inmuno.org (L.R.); sherrera@inmuno.org (S.H.); marevalo@inmuno.org (M.A.-H.)

In the original publication [1], the ethical approval code and date information was missing. This has now been revised to Section 2.1 “Study Design and Subject Recruitment”, the last sentence of the paragraph and added to the Institutional Review Board Statement at the Back Matter of the paper.

The corrected statement appears below. 

“All patients provided written informed consent for participation before enrolment, and the study protocol was approved by the Institutional Review Board (IRB) for the Malaria Vaccine and Drug Development Center (CECIV Approval letter 1.0) with DMID protocol 13-0056 on 14 February 2014, also amended on 1 October 2014 (Version 2.0) and 27 January 2016 (Version 3.0). In addition, the Georgia Institute of Technology IRB provided subsequent approval for genomic analyses on 4 April 2014, Amendment #1 to protocol H13495.”

The Institutional Review Board Statement appears below.

**Institutional Review Board Statement:** The study was conducted in accordance with the Declaration of Helsinki, and approved by the Institutional Review Board for the Malaria Vaccine and Drug Development Center (CECIV Approval letter 1.0) with DMID protocol 13-0056 on 14 February 2014, also amended on 1 October 2014 (Version 2.0) and 27 January 2016 (Version 3.0). In addition, the Georgia Institute of Technology IRB provided subsequent approval for genomic analyses on 4 April 2014, Amendment #1 to protocol H13495.

The authors state that these corrections do not impact the overall findings and conclusions of the paper. This correction was approved by the Academic Editor. The original publication has also been updated.
